# Toxic milk mice models of Wilson’s disease

**DOI:** 10.1007/s11033-021-06192-5

**Published:** 2021-02-15

**Authors:** Krzysztof Hadrian, Adam Przybyłkowski

**Affiliations:** Department of Gastroenterology and General Medicine, Medical University of Warsaw, Banacha 1a Street, 02-097 Warsaw, Mazovian Voivodeships Poland

**Keywords:** Wilson’s disease, Toxic milk mouse, WD, Animal models, Hepatolenticular degeneration

## Abstract

Wilson’s disease (WD) is a rare genetic disorder inherited as an autosomal recessive trait. The signs and symptoms of this disease are related to dysfunctional ATP7B protein which leads to copper accumulation and cellular damage. The organs that are most commonly affected by WD are the liver and brain. The dysfunctional ATP7B homolog has previously been identified in many different species, including two naturally occurring murine models called toxic milk mice. The aim of this paper was to compare the toxic milk mouse described by Rauch (tx) to that from Jackson Laboratory (txJ) through a review of studies on these two groups of mice. The two mice strains differ in the type of carried mutation and the phenotype of the disease. The data of the studies showed that the tx mice developed mild chronic hepatitis but suffered severe organ destruction with faster progression to full-liver cirrhosis. No changes were noted in the neurological and behavioral status of this strain despite the described toxic accumulation of copper and neuronal destruction in their brain. On the other hand, though the Jackson toxic milk mice (txJ) also presented chronic hepatitis, the condition was a bit milder with slower progression to end-stage disease. Moreover, hepatocyte suitable to perform neurobehavioral research as their phenotype characterized by tremors and locomotor disabilities better corresponds with the cliniconeurological picture of the humans.

## Introduction

Hepatolenticular degeneration, also known as Wilson’s disease (WD), was described for the first time in 1912 by a British neurologist, Sir Samuel Alexander Kinnier Wilson [1]. This genetically determined disease develops as a result of dishomeostasis of copper, an element that plays a major role as a cofactor in metabolic pathways. Copper deficiency leads to serious developmental disturbances. The amount of copper ingested with food and absorbed through the intestinal wall highly exceeds the demand of an organism. In healthy individuals, ATP7B protein, a specialized P-type ATP-ase, regulates and maintains the proper concentration of copper through its excretory activity. It is a transmembrane protein with several characteristic regions necessary for transport. Its cytosolic N-terminal domain can bind up to six Cu^+^ ions [2]. Mutation of the *ATP7B* alleles, which is the genetic background of WD, results in the formation of a defective protein leading to ineffective biliary excretion of copper and impaired ceruloplasmin synthesis (a major copper-transporting serum protein) [3]. Accumulation of Cu, especially in the liver, brain, and kidneys, is linked to various organ-specific symptoms of WD.

### Pathophysiology

Copper homeostasis is complex and depends on the cell type. It is regulated at many levels (eg. membrane trafficking, storage, kinase activity, DNA or RNA binding, etc.) and can vary in the arrangement of regulatory and binding protein pathways between different tissues. The majority of copper uptake pathways depend on the copper transport receptor 1 (Ctr1) protein [4]. The unbound copper is sequestrated by metallothioneins (MTs) and glutathione or connected with cuproenzymes by specific chaperones such as Atox1, copper chaperone to superoxide dismutase (CCS), Cox17, Cox11, Sco1, and Sco2. Atox1 is related to ATP7A and APT7B, CCS delivers ions to superoxide dismutase, and others mediate copper incorporation into mitochondrial cytochrome C oxidase complex [5]. In the liver, a major copper excretory organ, copper is involved in the holo-ceruloplasmin synthesis. For both the delivery of copper to apoceruloplasmin and excretion of copper to bile, the activity of ATP7B protein is crucial. In WD, inherited dysfunctional ATP7B is responsible for the accumulation of copper in the liver, oxidative stress, and cellular damage that triggers inflammation. This may result in acute liver failure or fibrosis often progressing to cirrhosis, while in the case of the brain, this condition may result in the development of neurological and psychiatric symptoms. Excess of copper is, due to high reactivity, involved in the production of cytotoxic reactive oxygen species (ROS). Because of the incontrovertible role of copper in the cellular oxygen respiration (as a cofactor of cytochrome C oxidase), alteration of mitochondrial structure and function is observed in the hepatocytes of WD patients in the early stages of the disease [6, 7]. Another manifestation of toxic overload is lipid oxygenation as an effect of higher concentrations of ROS [8]. Both changes result in inappropriate energy management and decreased cholesterol synthesis, thereby leading to hepatic steatosis. Oxidative stress is also a cause of nucleic acid instability which increases hepatocyte apoptosis and the risk of neoplastic transformation [9].

### Epidemiology

The prevalence of WD was estimated for the first time in 1968 as five individuals per million [10]. However, further investigations showed different numbers in various geographical regions. At one of the extremes, the data presented by Cocos described that the population of the Rucar region in Romania had the highest ever prevalence estimated (885 per million) [11]. The actual prevalence of WD remains to be elucidated. The birth prevalence of the disease is currently estimated to be about 30 per million worldwide, but because of the complex and modifiable characteristics of the disease (eg. environmental, genetic, epigenetic, and metabolic factors), researchers who apply genetic sequencing methods for estimation have reported that the prevalence may be even higher than 150 per million [12].

### Genetics

WD is an autosomal recessive inherited disorder. Petrukhin et al. identified the affected region in the genome of WD patients [13, 14]. It has been found that mutation in both alleles encoding the P-type ATP-ase ATP7B results in a defective protein, while mutation in only one allele causes mild, clinically nonsignificant aberration in copper metabolism [15–17]. Since 1993, more than 700 mutations of the *ATP7B* gene have been identified [18]. A substantive number of patients have different mutations in each copy of their chromosome 13 (compound heterozygotes). The most common mutation variant responsible for WD in up to 40% of the affected individuals worldwide is histidine-to-glutamine substitution at amino acid 1069 (p.H1069Q). This mutation is often found in the population of central and northern Europe and also that of the USA. By contrast, the Asian population has a more complicated distribution of mutations with no such clear presentation [19]. Because of these variations in mutations and modification factors, the clinical picture of the disease is considered to be highly variable. Significant differences in phenotype can even be noted in human offspring carrying the same mutations [20], and no evidence of universal and clinically useful genotype–phenotype correlations has been found despite numerous encouraging publications.

### Clinical picture

Symptoms of WD can occur at any time between early childhood and old age. They may include mild-to-aggressive and recurrent hepatitis, fulminant hepatic failure, hemolysis, and neurological or psychiatric symptoms such as an akinetic–rigid syndrome similar to Parkinson’s disease, tremor, ataxia, dystonic syndrome, insomnia, seizures, depression, anxiety, and psychosis [21, 22]. Corneal deposits of copper, often observed in the patients with neurological manifestation of WD during clinical examination, had been described by Kayser and Fleischer 10 years before the original article of Wilson was published [23]. For a long time, “Keiser–Fleischer rings” were considered as pathognomonic of WD. However, it has now been confirmed that these rings can also be seen in patients with chronic cholestatic diseases [24]. Depletion of ceruloplasmin in patients’ serum is one of the major biochemical features of WD. It was Holmberg who first described the function and role of ceruloplasmin in copper metabolism [25]. Estimation of nonceruloplasmin copper concentration in serum was proposed as one of the diagnostic tests for WD [26]. Another parameter that reflects copper metabolism is 24-h copper excretion in urine, which is significantly increased in Wilsonian patients. It was introduced into the diagnostic scheme in the late 60 s, but the precision of its cutoff is still under debate [27–29]. Hepatic biopsy samples are helpful in disease diagnosis but are not considered necessary. The histopathologic findings are nonspecific for WD, but the content of copper in these samples may confirm the diagnosis especially when analyzed together with other biochemical parameters. Genetic tests are the best choice and give the most certain result in diagnosis and familiar screening.

### Animal models of Atp7b dysfunction

Animal models are commonly used for studying the pathophysiology of human diseases. The following species express dysfunctional ATP7B protein homologs and share similarities with WD: Long–Evans Cinnamon (LEC) rat, LPP Atp7b^−/−^ rat, toxic milk mice, and knockout Atp7b^−/−^ mice. The majority of WD patients have missense mutations similar to the naturally occurring murine models which have a better corresponding phenotype, and thus, mice are generally preferred for the research on human diseases. In particular, liver inflammation, steatosis, and fibrosis are better represented in inbred mice strains. Knockout mice are alternative murine models and are genetically engineered by introducing several early terminating codons in the exon 2 of *Atp7b.* LEC rats have a spontaneous partial deletion in *Atp7b*, and three independent mutations have been identified so far [30]. These rats present low apoptotic status and high mitotic potential with DNA accumulation, which predispose to hepatocarcinogenesis [31]. LEC rats also present neurological and behavioral impairment which is not identical to humans but close enough to perform studies about the neurological pathology of WD [32]. LPP Atp7b^−/−^ rats were generated by crossing the LEC rats with the Piebald Virol Glaxo strain. In contrast to the LEC rats, LPP Atp7b^−/−^ rats do not exhibit the cinnamon fur color or die because of fulminant hepatitis about 3 months after birth. Both LEC and LPP Atp7b^−/−^ rats do not produce copper-deficient milk. Their pups do not require cross fostering which is similar to the situation in human development. In this review, we compare the two naturally occurring murine models of WD: the tx model described by Rauch in 1983 and the txJ model described by Jackson Laboratory.

## Survey methodology

Research papers related to toxic milk mice, published in English from 1950 to 2019, were searched in the Web of Science database. The following 11 search terms were used for the query: “Toxic AND MILK AND (Mouse OR Mice) OR Tx OR TxJ OR Tx-J OR TxR or Tx-R” and “Wilson* AND Disease AND (Mouse OR Mice).” An initial search resulted in 2969 articles. The titles, abstracts, and key words of the articles were further examined to judge their relevance. Finally, a total of 49 rodent studies that were highly relevant to the topic were included in this review.

## Toxic milk mouse described by Rauch

### History of discovery

In 1983, Rauch described the spontaneous mutants of the DL-strain mice which were characterized by the following phenotype: reduced pigmentation, poor growth, tremors, abnormal locomotor behavior, and ultimately death at 2 weeks of age [33]. Precise investigations showed that cross fostering of the affected infants by other (healthy) dams resulted in milder symptoms in litters and enabled them to survive, and the mice showed normal fertility in adulthood. Professor Rauch had been observing this mutated strain since 1974. Because of the lethal influence of breastfeeding by mother, Rauch designated this new mutant strain as toxic milk (tx). Different types of crossing suggested that this condition is transmitted by recessive autosomal inheritance. Rauch and his team accurately suspected that the mutants had copper metabolism disorder. They also found much evidence to prove their thesis about the role of copper overload in diseases of the liver and brain but were not able to determine the affected gene. The first evidence was the identification of a low concentration of copper in tx dam milk in the stomach content of 4- and 8-day-old pups. Another significant evidence was the fast reversal of the symptoms of “tx-infant syndrome” after few (2–3) injections of CuSO_4_. Oral supplementation of a copper solution also prevented health decompensation in the animals. Further investigations by other researchers showed that the homologous sequence to the human *ATP7B* gene probably lies near the centromere of murine chromosome 8 [34, 35]. However, the exact genetic background had remained unclear until 1996 when Theophilos identified a spontaneous, single mutation in the coding sequence of WND. Substitution of methionine 1356 in the eighth transmembrane domain by valine leads to an amino acid missense in the highly conserved Atp7b protein. This missense mutation results in the loss of the copper transport function of the protein causing pathological changes in the murine model of WD [36].

### Milk toxicity

The phenomenon of milk toxicity was clarified by Michalczyk et al. The authors analyzed the function and role of the normal and mutant *Atp7b* gene in the mammary gland in their study. They concluded that high copper intake results in a higher concentration of copper in the mammary gland of tx mice and in a minor redistribution of WD protein from the perinuclear region to the cytosol compartment, but without exerting any influence on the copper level in milk [37]. In the liver, brain, and skin tissues of 4- and 8-day-old tx infants, the copper levels, measured by atomic spectroscopy, were found to be extremely low. Survival of the control DL-strain pups that were fostered and breastfed by mutant mothers proved that the development of disease symptoms was not just triggered by copper-deficient milk. It became clear that intrauterine conditions during pregnancy and placental functions are crucial to facilitate adequate storage of copper necessary during early infancy [33]. Later investigations by another group of authors led to the discovery that Atp7b transporter responsible for the efflux of bile copper is also involved in the secretion of copper in proper amounts from mammary epithelial cells to milk. By contrast, Atp7a was found mainly at the basal part of the cell membrane, committed to the reabsorption of copper from the cytosol into the bloodstream. Both transporters were also found to be present in the fetal and maternal sides of syncytiotrophoblast and strictly regulate copper transport during gestation [38]. Breakdown of the development of the central nervous system and metabolic instability due to copper deficiency were suggested as the direct causes of the death of toxic milk mouse pups. This phenomenon of copper deficiency in milk and extremely decreased copper concentration in the organs afterbirth was never described earlier in humans suffering from WD [39].

### Aberration of metal metabolism

The concentration of copper measured in the liver of 7-week-old toxic milk mice was 10 times higher than that determined in control wild-type DL strain. Serum concentration of copper and ceruloplasmin was found to be decreased by about 50% in these animals [33]. Data published by Howell et al. indicated that all the organ tissues (liver, brain, spleen, kidney, femoral muscle) and blood samples (red blood cells and serum) obtained from 5- to 15-month-old tx mice had significantly elevated copper concentrations. Zinc level was also elevated in most of the samples, with significant differences noticed in the liver, kidney, and brain tissues [40]. Allen analyzed tx mice of five different age groups (5 days, 2–4 months, 5–7 months, 8–11 months, 12–21 months) and reported that the results were comparable to those presented in 1983. The concentrations of copper and iron in 5-day-old infants were significantly lower than those in controls. Older tx groups were distinctly copper overloaded. A different situation was observed for the metabolism of iron, the concentration of which reached normal values after 2 months and was consistently maintained at this level later. Zinc content in the liver at birth was similar to that of controls but kept increasing with age and reached about a twofold higher value. Ion concentrations were not significantly different between the copper-loaded tx and control mutants, as similar to the histopathologic characteristics of their liver [41]. Independent of the liver protection machinery, the concentrations of Cu and Zn were similar in the samples taken from regenerative nodules as well as residual parenchyma [42]. While the copper concentration of the liver dramatically increased with age, other organs, such as the brain and kidneys, began to accumulate an excess of this metal with some delay.

### Liver pathology

The tx mutation [33–35] was described to cause loss of copper transport function and disturb copper-dependent trafficking of Atp7b from the trans-Golgi network, which is crucial for the excretion of excess Cu [43, 44]. Studies showed that morphologic changes were most evident in the livers of mice in those regions that were not affected by nodular remodeling. The authors highlighted the disproportion and enlargement of hepatic nuclei as well as the presence of many heterogenic intranuclear inclusions. Some uncommon presentations reported by them were necrotic hepatocytes, with only a few patches of necrosis. The tissue surrounding regenerative nodules contained increased numbers of inflammatory response cells and fibroblasts. Morphology of cells and parenchyma inside the nodules was similar to that seen in the livers of controls [40]. In another study, hematoxylin–eosin staining showed changes that were similar to the above-mentioned ones, with the exception of the early proliferation of bile duct and induction of oval cells. Cholestasis and fibrosis were rarely seen, and there was no evidence of hepatocellular carcinoma lesions even in the copper-loaded mice which survived for more than 2 years [41]. Morphologic, histologic, and ultrastructural changes (liver nodular remodeling, hepatocytic swelling, and necrosis) were found to progress, despite the fact that the plasma level of copper tended to decrease in mice older than 6 months. In age-advanced mice, massive differences in morphologic integrity were observed between regenerative nodules and the intervening liver parenchyma [45]. Significant changes were noticed in nuclei and endoplasmic reticulum of the affected degenerating hepatocytes. In addition, changes in ultrastructure such as accumulation of microvesicular lipid droplets together with deformed and enlarged mitochondria containing inclusions were spotted under a transmission electron microscope [46]. In 1998, Deng examined an older population of 11- to 12-month-old tx. He observed that in mutants, the macroscopic regenerative nodules composed of hepatocytes that had a normal histologic appearance, on the surface of the liver. Unlike nodules, residual liver parenchyma looked microscopically abnormal with large, atypical hepatocytes. However, both the samples taken from nodules and residual tissue had significantly elevated concentrations of MT and higher numbers of apoptotic cells. MTs are a group of low-molecular-weight proteins synthesized and released in response to heavy metal toxicosis. Their affinity to Zn and Cu and sensitivity of their gene expression to multiple agents (eg. intoxication, hormones, irradiation) suggest that they play a major role in the divalent ion homeostasis. Immunohistochemical staining showed intense signals from nuclear and cytoplasmic MT in normal and atypical hepatocytes compared to only cytoplasmic MT present in the control DL group. The author and his team concluded that the large deposits of Cu-MT complex seen in the affected liver may have genotoxic influence and result in intensified apoptosis [42]. In a study on 3- to 5-month old tx and wild-type control mice, Koropatnick determined the levels of MT mRNA in the liver 6 h after cervical dislocation. After 24 h of sacrificing the animals, he measured the concentration of posttranslational (MT) protein which was found to be 100-fold higher in the mutant group [47]. No significant differences were noticed in the level of MT in the liver between tx males and females. By contrast, the levels of copper in hepatic cytosol were up to 130-fold higher compared to controls, with significant sex differences between tx males and females. Hepatic accumulation of zinc was also higher in the mutant population but was not as significant as copper levels and with no sex-related differences. These results suggest that the high accumulation of MT in the liver in mutant mice is a very significant defense mechanism against the increase in copper concentration.

### Neuropathology

Rauch observed the behavioral abnormalities of the tx offspring as early as 1 week after their birth. Initially, minor body tremors occurred, but their intensity gradually increased and subsequently advanced to gravity lateral shaking. Furthermore, the mice showed difficulty in locomotion, with overbalancing and falling over, and exerted great efforts in righting themselves. Often, their forefeet were seen fisted and wrists flexed, while hindlimbs appeared paralyzed. Besides those abnormalities in the newborn tx mice, which could be considered as a result of copper deficiency, no major neurological symptoms, as seen in WD patients, were evident [33]. However results of recent studies showed significant tx behavioral impairment especially in: spatial learning and memory (Morris Water Maze Test), locomotor coordination (Pole Test), neurological deficits (Traction Test) [48].

Cu concentration was elevated by 20% in the brain of copper-loaded tx mice of all age groups (described earlier) in comparison to the nonloaded group. However, no change in zinc or iron concentration was observed in brain tissue during the experiment [41]. Another study showed that in 16-week-old tx mice, the free copper concentration in brain tissue elevated a few days after penicillamine (PCA) administration, which may lead to enhanced oxidative stress and increased destruction of neurons [49]. Role of cardiovascular injury influence on cerebral blood circulation described in WD and still needs to be completely clarified. Tx model allow to present cerebrovascular damage with endothelial edema and degeneration in 8–10 weeks mice. Results of the study identified positive ICAM-1 and VCAM-1 immunohitochemical reaction in brain microcirculation staining. Also expression of von Willebrand factor, thrombomodulin and anti- cardiolipin antibody was significantly elevated in tx group [50]. Both hepatocytes and neurons are high metabolic cells. Mitochondrial destruction can play essential role and may be the major link between background of hepato- and neurological pathology. As suggested process of mitophagy, normally occurring to stabilize mitochondrial quality and quantity, has significant influence on hippocampal copper induced damage [48].

### Treatment studies

A study involving intrasplenic liver cell transplantation showed that hepatocytes obtained from healthy 6- to 8-week-old DL males can proliferate and correct the copper metabolism disorders in 3- to 4-month-old tx mice. The results determined 4 months after the procedure indicated a significant reduction of copper level in the liver, kidney, and spleen in tx infants [51]. In 2007, Buck et al. performed a trial in which intrahepatic bone marrow stem cell transplant was carried out to correct liver function and reduce the level of copper in hepatic tissue. In their trial, 3- to 4-month-old mice were irradiated with a sublethal dose of 4 Gy, and after 1 day, the animals were subjected to the transplant procedure. The concentration of copper was measured 5 and 9 months after the transplant. The results determined after 5 months showed a significant decrease in the hepatic level of copper, but the final outcome did not confirm the long-term maintenance of disease correction, despite the presence of graft cells. Similar improvement was noted in the activity of ceruloplasmin oxidase, but it was also evident only for short term [52].

## Toxic milk mouse from Jackson Laboratory

### History of discovery

A mice strain with a similar phenotype as the tx mice described by Rauch was observed at the Jackson Laboratory (Bar Harbor, Maine), which is a commercially operating rodent housing and breeding facility. Effects of *Atp7b* mutation was noted in the inbred C3H/HeJ mice strain in 1987. This gene was later identified as responsible for such phenotype with diseases of the liver and central nervous system. Due to many similarities with Rauch’s tx in clinical characteristics, especially the harmful influence of natural breastfeeding, this strain was therefore named Jackson toxic milk (txJ). In txJ, the spontaneous point mutation occurs in exon 8, at position 2135, which leads to a G712D missense, predicted to be in the second transmembrane region of the Atp7b protein.

### Milk toxicity

Litters born to mutant females were found to be copper deficient and died before 2 weeks of age due to the consumption of copper-deficient milk. Similar to Rauch’s tx, txJ mice could survive if breastfed by healthy C3H dams. Milk toxicity disappeared in 5- to 6-month-old dams. Because of copper overload in the whole body, the milk of the mice probably contained minor amounts of copper, which were enough to restore the deficit in newborns. On the other hand, breeding attempts showed that txJ females older than 6 months had many problems with conceiving and in withstanding pregnancy independently of sires. Delivery was also a significant impediment to older animals and could be lethal to both offspring and mother. An additional breeding-limiting barrier was cross fostering in cases of preterm birth or sudden rejection of the mutant litters by the C3H dams (known to be good mothers). txJ mice were also very sensitive to any change in the environmental conditions, and the most crucial factor that should be pointed out is their aversion to noise and vibrations. Thus, depending on the surroundings, silencing devices or the ones emitting constant noise can be helpful when these animals are used in studies.

### Aberration of metal metabolism

The liver concentration of Cu, measured by atomic absorption spectrometry, was found to be elevated 30-fold after the first month of life and was up to 60 times higher than the normal values in mice sacrificed at the age of 3 months. During the next several months, the level of copper slightly decreased but still remained highly elevated during the first year [53]. Ceruloplasmin is one of the essential proteins involved in iron homeostasis with ferroxidase playing a crucial role in cellular iron efflux. Jonczy et al. conducted a 6-month experiment on txJ mice and found that the animals developed anemia because of functional iron deficit with decreased serum level despite copper overload in their liver. The authors demonstrated that the iron ions separated from toxins could not be efficiently used in erythropoiesis due to the impaired role of ceruloplasmin in iron homeostasis [54]. Bronson reported that the txJ animals exhibited copper overload in the thalamus and lentiform nucleus even at 3 months of age, but the toxic effect with significant neuronal injury was observed only in the 12-month old population [55]. In the brain stem and cortex of 12-month-old txJ mice, iron concentration was not elevated and was similar to controls [56]. Terwel et al. described the differences in copper distribution in each of the four brain structures in 12-month-old toxic milk mice. The copper concentration was observed to be increased in the cerebellum, striatum, and hippocampus, whereas in the cortex, no differences were seen in the deposits between those mice and the C3H controls [57].

### Liver pathology

In the txJ mice, liver degradation progresses rather slowly, with the full transformation from fibrosis to cirrhosis taking about 6–7 months. In a study on 2- and 12-month old mice, the serum concentration of ceruloplasmin, measured using a modified Ravin method, was found to be significantly lower in the mutant population [58]. In the older txJ population aged more than 10 months, the livers appeared pale in color and macroscopically irregular with regeneration nodules on the surface. Rhodanine staining was used to visualize the copper overload [57–59]. Histopathologic staining with hematoxylin–eosin showed enlarged hepatocytes containing nuclei of increased size with irregular nucleoli inside [57]. Organs collected from 4-month-old mice showed minor histopathologic changes under a light microscope, while the consecutive samples obtained after the next 2 months showed only increased inflammatory response. Electron microscopy showed changes in the shape and morphology of hepatic mitochondria after 3–4 months of age. Exacerbation of mitochondrial appearance was significant in the 6-month-old group with numerous pathologies such as dilated tips of cristae, increased density of matrix, and electron-dense inclusions [53]. Moreover, CD11b-reactive cells (macrophages and dendritic cells) were detected in the liver sections of the Jackson toxic milk mice. Elevated concentrations of IL-1β and TNF-α mRNA were reported to be related to chronic hepatitis. Because of increased serum levels of IL-5 and TNF-α, it was suggested that inflammatory reaction was not only confined to the liver but also spread throughout the whole body [57]. Roberts et al. examined the livers in 1- to 8-month-old txJ mice. The major abnormality reported was a significant (~ 25%) reduction in the activity of mitochondrial complex IV starting from the fifth month of life. The level of citrate synthase was primarily decreased in the youngest age groups, but eventually increased and reached a significant value by 5 months of age. The authors associated this observation with mitochondrial hyperplasia and progression of liver disease. Despite the increase in the activity of citrate synthase, no evidence of prominent depletion of mtDNA was found [53].

### Neuropathology

Only a few reports confirm the significant neuronal damage in txJ mice. In 1995, Bronson et al. published the results of their investigation regarding the impact of copper deficiency on brain structure. They examined 2- to 3-week-old male and female litters born to and fostered by their homozygous txJ/txJ mother. Tissue was isolated from pups in formol–acid–alcohol mixture, following which the brain was extracted and embedded in paraffin. Cross sections of the caudate nucleus, thalamus, midbrain, and cerebellum were prepared and stained with hematoxylin–eosin or cresyl echt violet and Luxol fast blue. The major finding from the stained sections was acute neuronal necrosis in the frontal, parietal, and occipital lobes. Temporal lobes were not affected in any of the samples. Necrotic changes were seen only in the deeper layers (3–6) of the neocortical sections. Lesions were scattered and found to be mixed with patches of healthy tissue. Three samples showed necrosis in the bilateral dorsal thalamus, but the rest showed no difference in the rhombencephalon and diencephalon. The severity of necrosis varied between the different brain samples, which also made it difficult to reach a clear conclusion [55]. Terwel et al. proved that neuroinflammation was marked in the striatum and corpus callosum, but not in the cortex and hippocampus in txJ mice [57]. Chan et al. did not find significant differences in proapoptotic markers suggesting low neuronal apoptotic activity in the neurons of 10-month-old txJ mice, in comparison with the healthy control group [60]. Magnetic resonance imaging scans showed demyelination in the lenticular nucleus, thalamus, and brainstem of 6- and 12-month-old txJ mice as well as decreased concentrations of myelin basic protein in the thalamus and lenticular nucleus in these animals [56].

For the first time, reports on motor and cognitive disorders in mutant mice from Jackson Laboratory were published in 2011. A study of 12-month-old txJ mice showed mild impairments in their performance in the rotarod test and their inability to acquire spatial memory in the Morris water maze. In the rotarod test, toxic milk mice acted differently compared to wild-type mice due to their preference for using forelimb, with maximal performance shown very slowly. Although copper deposition and inflammatory response observed in the striatum had a major impact on the motor behavior of these mice, it was observed that their spatial memory was even more impaired. Those results were very interesting since copper deposition and inflammatory response were much less evident in the hippocampus. The probable explanation for this observation was that copper deposition in the hippocampus impairs synaptic transmission, which could be responsible for the inability of these mice to acquire spatial memory [57]. Evidence in the literature supports the thesis that copper loading in rodents impairs long-term potentiation in the hippocampus [61]. Behavioral changes in Jackson toxic milk mice can result from copper-induced neuronal injury, but there may be other potential reasons as well. It cannot be eliminated that liver dysfunction is linked to increased production of toxic metabolites that disturb brain function as observed in WD patients with liver failure. In another study, impaired motor behavior, together with an increased level of copper and serotonin in different brain regions and a slight decrease of dopamine concentration in the striatum, was observed in 12-month-old Jackson toxic milk mice. Regardless of the liver disease, poor locomotor performance and physical condition of the aged toxic milk mice could be attributed to their faster aging [58].

### Treatment studies

In 2013, Medici et al. published the results of their trial comparing PCA and betaine (methyl donor) treatments. Subgroups of 12-week-old txJ and C3H control mice were treated with PCA or betaine for the next 12 weeks. Administration of oral PCA reduced the serum level of alanine aminotransferase together with the concentration of Cu in the liver by ~ 50% and the expression of *Tnf-α* gene in the organ by around 90% in the mutant group. In addition, mononuclear infiltration was improved in lobular and portal areas. By contrast, betaine only lowered the liver concentration of copper in the C3H control group and reduced the lobular inflammatory response. Both PCA and betaine had no effect on hepatocytes, especially the histologic appearance of their nucleus. However, the connection between decreased global DNA methylation, inflammatory response, oxidative stress, and lipogenesis in the untreated txJ subgroup remained unclear [62]. Other reports suggest a crucial role for choline supplementation during gestation and lactation in the epigenetic modification of fetal DNA expression which results in better general condition (body weight), and improved liver function with lower hepatic concentrations of Cu and Fe in 3-week-old litters. Maternal choline modifies liver concentration of copper in the fetus as well as gene expression, DNA methylation, and neonatal growth in the txJ mouse model of WD [63].

## Atp7b^−/−^ knockout mouse

### History of discovery

In 1999 concerns about influence of tx and txJ mutations on expression and function of Atp7b protein push Buiakova et al. to generate completely new strain of homozygous mutants (null) for the WD gene. Substitution of portion of Atp7b mRNA with early termination codon in exon2, followed by homologous recombination to avoid intron- exon boundaries was designed to stop the translation in every of 3 possible reading frame [64]. In result of their work there was created genetically engineered organism to be third murine model of the disease with clear origin and molecular background.

### Milk toxicity

Pups are usually borne smaller than normal wild- type and significantly copper deficient. Natural breastfeeding by dams producing copper deficient milk can be lethal to part of newborns. Despite the fact of very low copper concentration in Atp7b^−/−^ milk the ‘milk toxicity’ phenomenon do not appear in first generation of this strain born to heterozygous parents. Authors described symptoms of ‘infant syndrome’ corresponding to this present in toxic milk strains in some of pups born to homozygous parents in second generation. Cross-weaning was not performed but nursing by Atp7b^−/−^ mother results in noticeable slower and worst development of newborns. The multi organ Cu deficit developing during pregnancy period is probably less severe than in both toxic milk strains. On the other hand better survival results can be present due to more efficient copper intestinal absorption or better function of other pathways regulating copper metabolism [64].

### Aberration of metal metabolism

Similarly to both toxic milk strains, *Atp7b* null mice start with very low hepatic copper concentration determined by polarized absorption spectrometry in dry tissue. In effect of introduced genetic modifications lack of Atp7b as suspected leads to rapid liver copper overload described in 2 month mutants. Analysis of copper concentration show copper overload in placenta and lactating mammary gland but lack of ATP7B activity makes maternal–fetal Cu transport and exocrine function insufficient [64]. Observation of intracellular copper concentration showed nonuniform distribution, especially in cytosol and inside nucleus [65].

### Liver pathology

Adult Atp7b^−/−^ mice due to obvious accumulation of liver copper deposits exhibit a more severe phenotype with metallothionein overexpression, much faster progression to liver cirrhosis and predisposition to intrahepatic neoplasia [65]. Such a phenotype, in comparison to toxic milk models, can make interpretation difficult in some cases and seem problematic because of disparity in the clinical manifestation of WD. Recent studies showed that loss of hepatocytic Cu deposits efflux mediated by Atp7b cause cuprotoxic damage to mitochondrial membranes and proteins. In result of which authors described massive leakage of proapoptotic factors and observed processes that convert mitochondria into autophagic sequestration. Intriguing fact is that inhibition of mitophagy with spautin-1 was connected with sequence of mitochondrial impairment and secondary serious hepatic dysfunction [66]. Another complication is lack of clear and simple description of influence of WD on metabolic status. Because of genetic variability and environmental influence unification of the patients’ metabolic profile is impossible. There are only few case reports regarding towards glucose tolerance in WD, with rather contradictory conclusions. Use of the genetically generated mice in metabolic research allow to observe increased glucose tolerance and insulin sensitivity together with defective counterregulatory response. As suggested improved glucose tolerance in the model mice may be an effect of progressive liver carbohydrate homeostasis dysfunction, rather than result of healthy metabolism [67]. Significantly lower concentrations of liver and serum lipids were noted in null mice together with downregulation of genes managing cholesterol and other lipid homeostasis [67, 68]. Atp7b^−/−^ seems to be very useful in further assessment of copper dependent lipid metabolic impairment.

### Neuropathology

The neurological signs and behavioral alterations in adult were not reported and are not well characterized in genetically engineered mice. In genetically based lack of brain fraction of Atp7b, it’s ceruloplasmin forming activity, probably can be compensated by upregulation of *atp7a* expression in surrounding glial cells, with higher concentrations of holoceruloplasmin. Authors suggested that this may explain such neurologic phenotype and absence of characteristic symptoms [69]. However some of reports from metabolic studies suggests that western diet fed Atp7b^−/−^ had better metabolic profile (reduced hepatic steatosis and reduced obesity) than wild type controls. As suggested that may be secondary to visibly higher level of their locomotor activity [67]. This presentation more similar to Toxic Milk mice described by Rauch does not match clinical picture of WD in which more than 45% of patients have neurologic disturbances. That fact can be problematic in design of further projects using null mice especially when investigators need to asses both neurologic and hepatologic degeneration.

## Conclusion

The described strains of toxic milk mice share many similarities, and both are approved animal models of WD (Table 1). Cu^2+^ was found to induce hepatic injury and modify the impact of this condition on the structure and activity of Atp7b protein, which were thoroughly documented and determined to be close enough in both murine models. Tx and knockout, mice seem to have a major hepatologic phenotype with faster progression of liver disease and worse prognosis. The locomotor disturbances described in less than 2-week-old tx pups by Rauch himself are very suggestive but rather correspond with copper deficiency in newborns which is unusual in human infants. On the other hand, the absence of movement disorders together with normal behavior in older tx mice is not in agreement with the neurological disturbances described for WD patients and txJ mice. These inconsistent findings suggest that txJ may serve as a better model in further neuropsychiatric research elucidating neurodegeneration in the background of copper toxicity.

**Table 1:** Short characteristics of mice models of Wilson’s disease

	Toxic milk mouse	Jackson toxic milk mouse	ATP7B−/− mouse
Origin	Natural inbred mutation	Natural inbred mutation	Genetically engineered
Mutation	Point mutation 1356 Met—> Val	Point mutation 712 Gly—> Asp	introduction of an early termination codon in exon 2 of mouse ATP7B mRNA
Natural disease course	Progression to full-liver cirrhosis until 1 year	Slightly slower progression to liver steatosis and cirrhosis	Earlier development of severe liver pathology than in natural inbred models
Earliest notable changes in the liver	Nodular fibrosis, bile duct hyperplasia, and inflammatory infiltration at 6 months	Increased inflammatory infiltration at 5 months Mild fibrosis at 6–7 months	Ultrastructural changes, steatosis, and mild inflammation at 6 weeks Hepatitis, dysplasia, and necroinflammation at 4–5 months
Liver morphology in end-stage disease	Hepatocyte steatosis, fibrosis, and progression to full-liver cirrhosis	Fibrosis with nodular regenerative remodeling, cirrhosis, and rarely HCC	Bile duct proliferation, fibrosis, neoplastic proliferation. Regeneration processes often seen in large parts of the liver
Behavioral, neurological, and locomotor changes	No evidence of neurological or behavioral disturbances reported	Light changes: forelimb usage preference and slower locomotor learning (rotarod)	No remarkable neurologic/behavioral symptoms or evident brain pathology were observed
Elevated copper concentration in the liver	** + **	** + **	** + **
Elevated copper concentration in the brain	** + **	** + **	** + **
Copper-deficient milk	** + **	** + **	** + **
Need of cross-fostering	** + **	** + **	**–**
Decreased ceruloplasmin concentration	** + **	** + **	** + **

*Met* methionine, *Val* valine, *Asp* aspartic acid, *Gly* glycine, *HCC* hepatocellular carcinoma

## Data Availability

No scientific data was used in the review.

## Code availability

No code was used in the review.
